# Increased Orbital Muscle Fraction Diagnosed by Semi-Automatic Volumetry: A Risk Factor for Severe Visual Impairment with Excellent Response to Surgical Decompression in Graves’ Orbitopathy

**DOI:** 10.3390/jpm12060937

**Published:** 2022-06-06

**Authors:** Christine Steiert, Sebastian Kuechlin, Waseem Masalha, Juergen Beck, Wolf Alexander Lagrèze, Juergen Grauvogel

**Affiliations:** 1 Department of Neurosurgery, Medical Center—University of Freiburg, Faculty of Medicine, University of Freiburg, 79106 Freiburg, Germany; waseem.masalha@uniklinik-freiburg.de (W.M.); j.beck@uniklinik-freiburg.de (J.B.); juergen.grauvogel@uniklinik-freiburg.de (J.G.); 2 Eye Center, Medical Center—University of Freiburg, Faculty of Medicine, University of Freiburg, 79106 Freiburg, Germany; sebastian.kuechlin@uniklinik-freiburg.de (S.K.); wolf.lagreze@uniklinik-freiburg.de (W.A.L.)

**Keywords:** orbital muscle volume, increased orbital muscle, orbital muscle enlargement, orbit volumetry, Graves’ orbitopathy, Graves’ disease, dysthyroid optic neuropathy, orbital decompression

## Abstract

Graves’ orbitopathy (GO) leads to increased orbital tissue and causes symptoms such as exophthalmos, functional complaints, or dysthyroid optic neuropathy. Different GO types with fat and/or muscle enlargement were identified, and increased muscle appears to particularly influence visual status and treatment response. The current study examines visual parameters dependent on orbital muscle volume fraction in a surgically treated GO cohort. After volumetric analysis of the preoperative orbital content, 83 orbits in 47 patients were categorized into predefined groups (increased or not-increased muscle fraction). All cases underwent pterional orbital decompression, and pre- and postoperative visual status was retrospectively analyzed. Forty-one orbits revealed increased and 42 orbits revealed not-increased muscle volume (mean fraction 29.63% versus (vs.) 15.60%). The preoperative visual acuity (VA) was significantly lower in orbits with increased vs. not-increased muscle volume (mean VA 0.30 vs. 0.53, difference 2.5 lines). After surgery, mean VA improved significantly by 1.7 lines in orbits with increased muscle volume. Not preoperative, but postoperative exophthalmos was significantly lower in orbits with not-increased muscle volume. Increased orbital muscle is associated with significantly reduced VA, but can be remarkably improved by pterional orbital decompression. Therefore, surgical therapy should be considered particularly in decreased VA with orbital muscle enlargement.

## 1. Introduction

In Graves’ orbitopathy (GO), a volume increase of orbital fat and/or muscle tissue due to an autoimmune inflammatory process leads to problems such as exophthalmos, double vision, and several functional complaints such as edema or ocular dryness [1]. The most severe complication is dysthyroid optic neuropathy (DON), where optic nerve compression causes optic nerve head swelling with the risk of consequent visual loss [2]. The therapeutic approach depends on the activity and severity of the disease [3]. Particularly in severe cases with DON, surgical decompression may be required [3,4]. Since prognosis in DON improves with early diagnosis and therapy induction, different clinical and imaging parameters have been studied to predict the risk of DON [5,6]. Imaging parameters focus on the analyses of the orbital content (e.g., the ratio of fat or muscle enlargement, the water fraction of the orbital tissue, or the content of the orbital apex (“apical crowding”)) [6,7,8,9,10]. Different types of GO were identified depending on the increase in orbital muscle volume and/or fat volume [1,11]. Increased orbital apex crowding has been found to be associated with DON, but overall increased orbital muscle volume does not seem to necessarily be related to DON [5,6,12,13]. However, orbital muscle volume appears to be of central importance, and respective studies have focused on predictors of DON [12,14].

To the best of our knowledge, there has been no investigation of the possible influence of increased orbital muscle volume on ophthalmological functional parameters or their development after orbital decompression. The present study is the first to determine the preoperative orbital muscle volume in a GO cohort, which were all treated subsequently in a standardized fashion (pterional orbital decompression), and to evaluate differences in preoperative ophthalmological status and postoperative ophthalmological outcome dependent on an “increased” or “not-increased” orbital muscle volume (following Regensburg et al. [11]).

## 2. Patients and Methods

### 2.1. Study Design and Patient Population

The inclusion criterion was pterional orbital decompression between January 2001 and May 2021 due to GO with the availability of preoperative cranial imaging suitable for volumetry (computed tomography (CT) (soft tissue window, slice thickness ≤ 2.0 mm) or magnetic resonance imaging (MRI) (three dimensional (3D) T1-weighted imaging (with or without contrast enhancement), or axial and coronal T1- or T2-weighted imaging, slice thickness ≤ 3.0 mm (if available, with fat saturation))). The indication for surgery was either the presence of DON as a sight-threatening condition (following EUGOGO classification [3]) with no or only insufficient response to high-dose steroid therapy, or surgery was performed due to severe functional complaints or cosmetic reasons in cases with moderate-to-severe disease severity (following EUGOGO classification). In detail, in cases with moderate-to-severe disease severity and active (following clinical activity score (CAS) [3]) disease, surgery was performed in individual exceptional cases with severe functional complaints persisting despite high-dose steroids (such as severe lid retraction > 2 mm or exophthalmos > 3 mm with corneal exposure and signs of developing corneal breakdown (cases with insufficient responsiveness to lubricants), or severe retrobulbar pain). In cases with moderate-to-severe disease severity and inactive (following CAS) disease, surgery was performed for cosmetic and functional reasons (e.g., to correct exophthalmos and lid retraction, to decrease intraocular tension, to improve preexisting strabismus, etc.) for rehabilitation purposes. All cases were discussed at the surgical board of our interdisciplinary center for orbital diseases.

According to the literature, DON was then considered to be present in our cohort if at least one of the following criteria was fulfilled in GO patients (for which no other underlying cause was apparent either): impaired color vision, reduced visual acuity, optic disc swelling/atrophy, visual field defects or relative afferent pupillary defect [1,15].

Pterional orbital decompression was performed in a standardized fashion as previously described [16]. Prior to the volumetric analysis with a focus on the influence of orbital muscle volume, which is currently being conducted for the first time, data on the postoperative outcome of some of the cohort had already been published [16].

The primary endpoint of the current study was whether there were differences in the preoperative status and the postoperative development of visual acuity and exophthalmos depending on the orbital muscle volume (“not-increased” or “increased” according to the described subtypes [11]). The secondary endpoints were to determine the distribution of the muscular subtypes within the whole group and within the subgroups of cases with and without DON, as well as to assess other factors having potential influence, such as clinical activity or disease severity.

The retrospective evaluation was based on medical records and radiological imaging. Every surgery was performed in the Department of Neurosurgery as a tertiary referral center. Informed consent for the surgical procedure was obtained from all patients or their legal representative. The retrospective analysis was approved by the independent ethics committee of our medical center (reference no. 351/19) and has been reported according to institutional guidelines.

### 2.2. Volumetric Analysis

**Figure 1:**
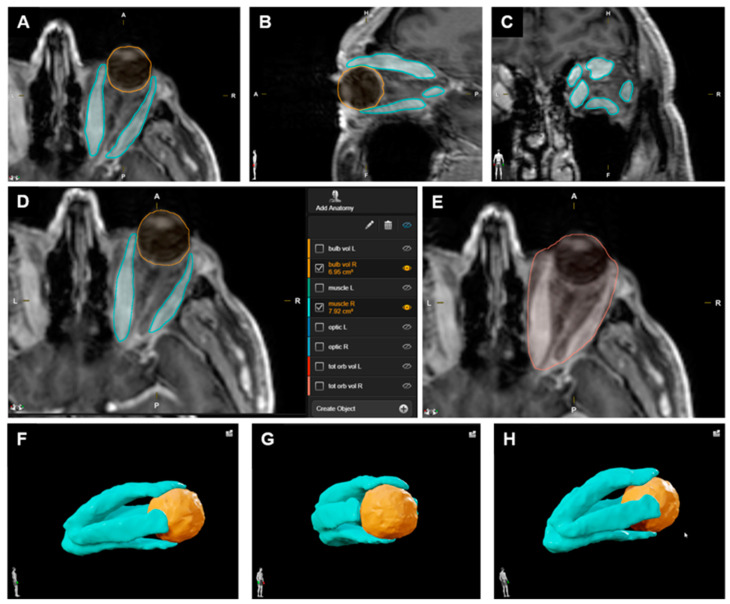
Illustration of the semi-automatic volumetry technique. (**A**–**C**): Volumetric analysis of the orbital content (right orbit) in a patient affected by Graves’ orbitopathy (GO) (not-increased orbital muscle volume) prior to decompressive surgery, with orbital extraocular muscles marked in blue and eye bulb in orange in an axial (**A**), sagittal (**B**) and coronal (**C**) view. (**D**): Absolute volumetric values (in cm^3^) provided by the system after three-dimensional (3D) analysis of the structures of interest (for example eye bulb with 6.95 cm^3^ and muscle with 7.92 cm^3^). (**E**): Volumetric analysis of the whole orbital content (marked in red in an axial view) for subsequent calculation of the orbital muscle fraction in relation to the total orbital volume. (**G**,**H**): 3D view of the volume-analyzed structures (orbital extraocular muscles marked in blue and eye bulb in orange), shown from different perspectives: lateral right (**F**), anterior oblique right (**G**), and posterior oblique right (**H**).

**Figure 2:**
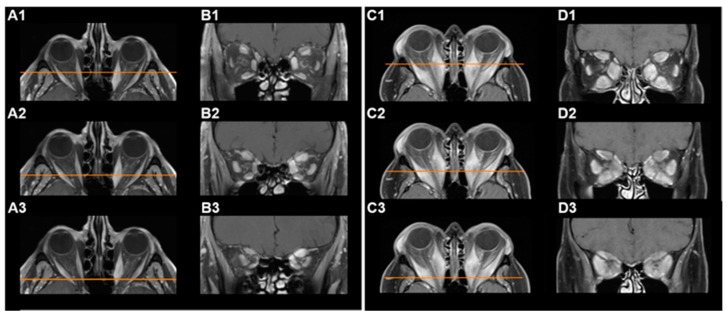
Magnetic resonance imaging (MRI) demonstrating not-increased or increased orbital muscle volume. (**A**,**B**): T1-weighted, contrast-enhanced MRI scans of the orbits of a bilaterally affected GO-patient with not-increased orbital muscle volume prior to decompressive surgery; axial images (**A1**–**A3**) with the orange line indicating the position of each corresponding coronal (**B1**–**B3**) image retrobulbar (**A1**,**B1**), at the orbital apex (**A3**,**B3**) and in between (**A2**,**B2**). (**C**,**D**): T1-weighted, contrast-enhanced MRI scans of the orbits of a bilaterally affected GO-patient with increased orbital muscle volume prior to decompressive surgery; axial images (**C1**–**C3**) with the orange line indicating the position of each corresponding coronal (**D1**–**D3**) image retrobulbar (**C1**,**D1**), at the orbital apex (**C3**,**D3**) and in between (**C2**,**D2**).

Volumetric analysis of intraorbital (extraocular) muscle volume and total orbital volume was performed in a semi-automatic technique with manual adjustment by two independent investigators using the SmartBrush device on the Brainlab Origin Server 3.2 (Brainlab AG, Munich, Germany), and the corresponding mean values were used for further analysis. Group classification was based on the calculated fraction of muscle volume relative to total orbital volume. The not-increased orbital muscle volume group (not-increased group) had a muscle fraction ≤ 0.19, and the increased orbital muscle volume group (increased group) had a muscle fraction ≥ 0.21 (following Regensburg et al., 2011 [11]). The volumetry technique using the Brainlab system is illustrated in Figure 1. MRI scans with examples of a not-increased and an increased orbital muscle volume are presented in Figure 2.

### 2.3. Ophthalmological Outcome

Preoperative ophthalmological data were obtained within the four weeks prior to surgery, and in acute sight-threatening cases, usually within the week prior to surgery. The measurement of visual acuity documented in the medical records in decimal notation was converted to the mean angle of resolution (logMAR) for analysis. It is important to note that better visual acuity correlates with lower logMAR values, and one line read on a standard vision chart corresponds to a difference of 0.1 logMAR. Exophthalmos assessments were performed by Hertel exophthalmometry. Within six months after surgery, the best documented values for visual acuity and exophthalmos were taken as postoperative values for analysis.

### 2.4. Statistical Analysis

Methods of descriptive statistics were used. Categorical data are presented as absolute and relative frequencies (in %). For numerical data, mean values with the minimum/maximum and the standard deviation (SD), standard error of mean (SEM) and confidence interval (CI) were calculated (as appropriate). Interrater reliability in volumetric analysis was validated using a Bland–Altman plot. Statistical differences were evaluated using a Mann–Whitney test as an unpaired nonparametric test. The level of significance was set to *p* < 0.05. Statistical analysis was performed using GraphPad Prism software version 9.1.1 for Mac (GraphPad Software, San Diego, CA, USA).

## 3. Results

### 3.1. Volumetry

**Figure 3:**
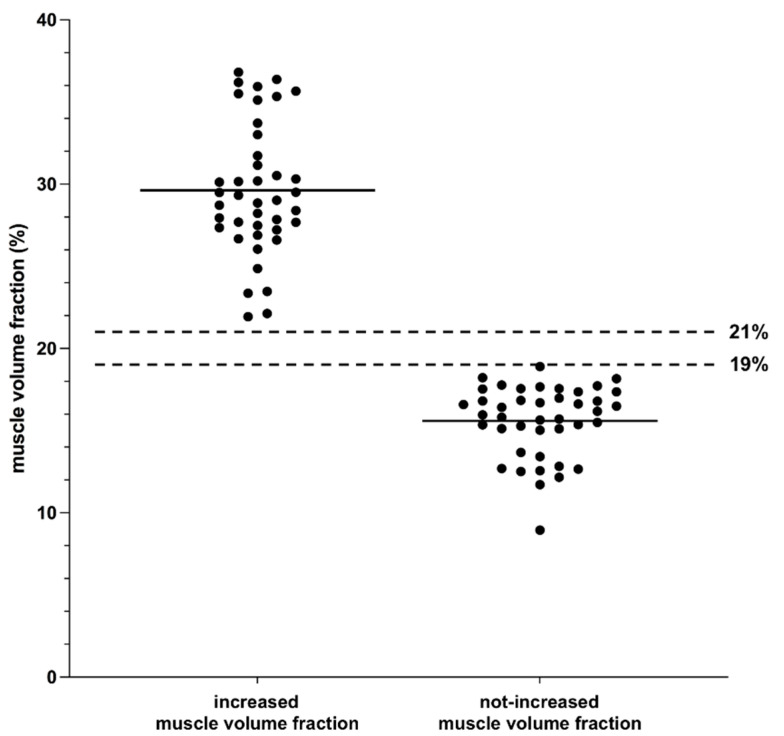
Volumetric analysis of orbital muscle volume fraction prior to orbital decompression. *y* axis: Orbital muscle volume fraction in relation to total orbital volume in %, *x* axis: group of orbits with an increased orbital muscle volume fraction (left, fraction ≥ 21%) and group of orbits with a not-increased orbital muscle volume fraction (right, fraction ≤ 19%), black dots: muscle volume fraction per orbit, horizontal black line: mean of values, dotted black lines: cut-off values (21% for increased muscle volume fraction and 19% for not-increased muscle volume fraction).

The analysis of orbital muscle volume in relation to total orbital volume was performed in 91 orbits in 54 GO patients. Two groups could be classified in accordance with a “not-increased” orbital muscle fraction (≤0.19) or an “increased” orbital muscle fraction (≥0.21). Due to having an orbital muscle volume fraction between 0.19 and 0.21, eight orbits in seven patients were excluded. A total of 83 orbits in 47 patients were included in the study with an equal distribution among the groups (not-increased group: 42 orbits; increased group: 41 orbits). The mean orbital muscle volume fraction in the increased group was 29.63% (range 21.94–36.81%, SD 4.00, SEM 0.62, CI 28.36–30.89), which was significantly higher than in the not-increased group with a mean orbital muscle volume fraction of 15.60% (range 8.94–18.90%, SD 2.16, SEM 0.33, CI 14.93–16.28), *p* < 0.0001 (see Figure 3). The number of cases that were affected by DON (sight-threatening condition following EUGOGO classification) did not differ significantly between the two groups (increased group: 80.49% with DON, not-increased group: 71.43% with DON). The gender ratio was unequal in the not-increased group, with significantly more women than men affected (29:13, *p* = 0.014), whereas there was an equal distribution in the increased group (23:18). There were no significant differences between the groups regarding age, affected side, or clinical activity score (CAS). Detailed information is listed in Table 1.

### 3.2. Outcome—Visual Acuity

**Figure 4:**
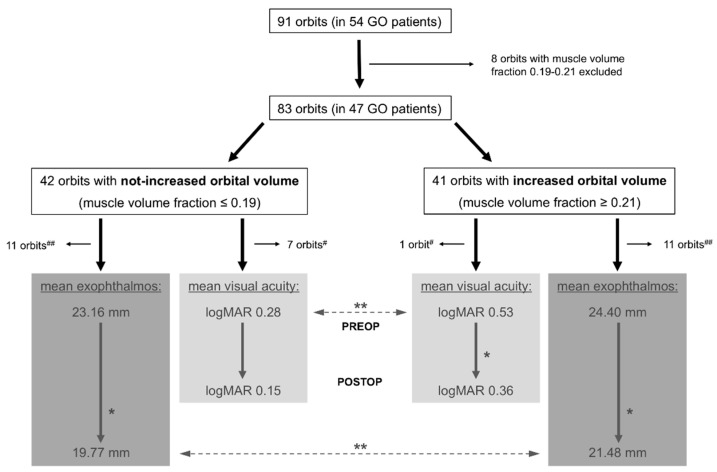
Inclusion criteria, group classification, and ophthalmological outcome. GO, Graves’ orbitopathy; ^#^, exclusion due to unavailability of postoperative assessment of visual acuity; ^##^, exclusion due to unavailability of postoperative assessment of exophthalmos; PREOP, preoperative assessments; POSTOP, postoperative assessments; logMAR, logarithm of the mean angle of resolution; *, statistically significant difference in intra-group analysis (comparison between pre-versus (vs.) postoperative results within the groups); **, statistically significant difference in inter-group analysis (comparison between preoperative or postoperative results between the two different groups).

**Figure 5:**
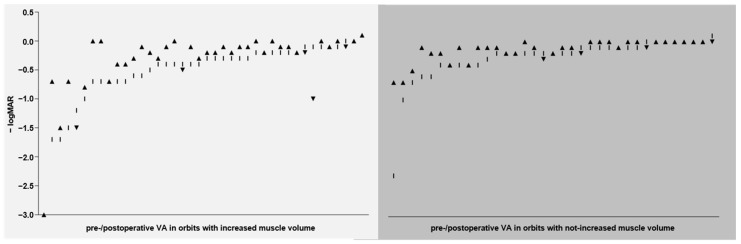
Development of visual acuity after orbital decompression. −logMAR, negative logarithm of the mean angle of resolution; VA, visual acuity; *y* axis, VA values per orbit before and after orbital decompression (given as negative logMAR, which means that the more negative the value, the worse the visual acuity); *x* axis, pre-/postoperative VA values of each orbit in the group with increased orbital muscle volume (left, light gray background) and in the group with not-increased orbital muscle volume (right, medium gray background); vertical line, preoperative VA value per orbit; black triangle (sharp angle upwards), postoperatively improved (or stable) VA value per orbit; black triangle (sharp angle downwards), postoperatively deteriorated VA value per orbit.

Pre- and postoperative visual acuity measurements in accordance with the inclusion criteria were available for 35 of the 42 orbits in the not-increased group, and for 40 of the 41 orbits in the increased group. The mean preoperative visual acuity in the increased vs. the not-increased group was significantly reduced by 2.5 lines (logMAR 0.53 vs. 0.28, *p* = 0.0058). The mean postoperative visual acuity in the increased vs. the not-increased group was reduced by 2.1 lines, but without statistically significant difference. Within the increased group, the mean postoperative visual acuity was significantly improved by 1.7 lines compared with preoperative values (logMAR 0.36 vs. 0.53, *p* = 0.0138). Within the not-increased group, the mean postoperative visual acuity was improved by 1.3 lines compared with preoperative values, but without statistically significant difference. For a schematic illustration, please refer to Figure 4 and Figure 5. A potential influence of the severity of proptosis on preoperative visual acuity was also analyzed in the cohort, but no relevant effect on visual acuity related to the severity of proptosis could be observed.

A total of 28 orbits in the not-increased group and 32 orbits in the increased group were affected by DON. Within the DON cohort, the mean preoperative visual acuity in the increased vs. the not-increased group was significantly reduced by 2.9 lines (mean logMAR 0.63 vs. 0.34, *p* = 0.0035). The mean post- vs. preoperative visual acuity was significantly improved in both the not-increased and the increased group affected by DON (not-increased group: logMAR 0.18 vs. 0.34 (1.6 lines, *p* = 0.0447), increased group: logMAR 0.43 vs. 0.63 (2.0 lines, *p* = 0.0142)). In the DON cohort, the mean postoperative visual acuity in the increased vs. the not-increased group was reduced by 2.5 lines without a statistically significant difference. Further information is given in Table 2.

### 3.3. Outcome—Exophthalmos

**Figure 6:**
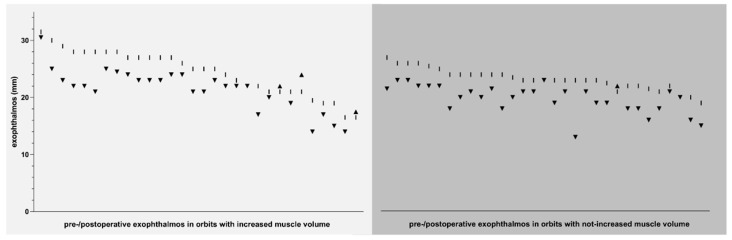
Development of exophthalmos after orbital decompression. *y* axis = exophthalmos values (in mm) per orbit before and after orbital decompression, *x* axis = pre-/postoperative exophthalmos values of each orbit in the group with increased orbital muscle volume (left, light gray background) and in the group with not-increased orbital muscle volume (right, medium gray background), vertical line = preoperative exophthalmos value per orbit, black triangle (sharp angle downwards) = postoperative decrease in exophthalmos (or stable exophthalmos) per orbit, black triangle (sharp angle upwards) = postoperative increase in exophthalmos per orbit.

Pre- and postoperative exophthalmos measurements in accordance with the inclusion criteria were available for 31 of the 42 orbits in the not-increased group, and for 30 of the 41 orbits in the increased group. The mean postoperative exophthalmos of the not-increased vs. the increased group was significantly lower (a difference of 1.71 mm, *p* = 0.0072), whereas there was no statistically significant difference between the corresponding preoperative values (a difference of 1.24 mm). Both groups showed a significant reduction in their mean post- vs. preoperative exophthalmos (not-increased group: a difference of 3.39 mm (*p* < 0.0001); increased group: a difference of 2.92 mm (*p* = 0.0082)). For a schematic illustration, please refer to Figure 4 and Figure 6.

The 24 orbits in each of the not-increased and the increased group affected by DON also presented a significant reduction in the mean exophthalmos postoperatively compared with the corresponding preoperative values (not-increased group: a difference of 3.55 mm (*p* < 0.0001); increased group: a difference of 3.13 mm (*p* = 0.0309)). In these orbits affected by DON, there were no significant differences between the not-increased and increased group regarding their mean pre- and postoperative exophthalmos values. Further information is given in Table 3.

### 3.4. Other Outcome Parameters

Diplopia was postoperatively completely resolved in 34.48% of cases in the increased group and in 14.29% of cases in the not-increased group. This difference did not reach statistical significance. New diplopia occurred postoperatively in two cases in the not-increased group (7.14%). There was transient headache after five operations (6%), periorbital hyposensitivity after two operations (2.4%) and clinical hemorrhage not affecting VA without further need for surgical treatment after one operation (1.2%).

## 4. Discussion

The visualization and analysis of the enlarged (intra-)orbital tissue in GO is of high importance in the understanding of the disease, particularly in severe cases and for the potential prediction of DON. Although MRI is also used for imaging diagnostics today, CT continues to be frequently used as the preferred technique since both the bony orbit and intraorbital structures are excellently visualized [1,6,17]. Volumetric analysis of (intra-)orbital structures has been established on the basis of both CT and MR images with comparable results [8,14,18,19,20]. In addition to fully manual segmentation (planimetry), the semi-automatic technique (with the possibility of manual adjustment) has also proven successful for volumetric analysis [21,22].

In studies of the natural disease course in untreated GO patients, fat enlargement tends to be classified as a late phenomenon, and muscle enlargement is rather associated with more severe disease activity [7,10,12]. An increased muscle volume in relation to total orbital volume was defined as a muscle fraction of 0.21 or more [10,11]. Regensburg et al., described four subtypes of GO based on CT volumetric analysis of 95 untreated GO orbits (25% without fat/muscle increase, 5% with only fat increase, 61% with only muscle increase, 9% with both fat and muscle increase) [11]. Thus, 69.5% of these orbits showed muscle enlargement with a mean muscle fraction of 0.24–0.25 (relative to total orbital volume), whereas orbits with “not-increased muscle volume” had a mean muscle fraction of 0.16–0.17. In our analysis with analogous definitions of “not-increased volume” and “increased volume” there was a highly significant difference between the group with “increased” mean muscle volume (fraction 0.29–0.30) and “not-increased” mean muscle volume (fraction 0.15–0.16). For healthy control groups, data on orbital muscle fraction range from 0.12 to 0.15 [7,23].

To the best of our knowledge, the current study is the first to examine orbital muscle enlargement as a potential factor that influences functional outcome in a large cohort of GO patients who were all treated with the same surgical approach. The choice of the approach for orbital decompression is discussed controversially in the literature. Despite a number of systematic reviews and also prospective studies, no general recommendations on the most suitable surgical procedure have yet been established due to various surgical indications and the heterogeneity of applied techniques. In addition, surgeons of different specialties have varying preferences depending on their personal skills and experience [24,25].

In our study, there was a balanced distribution of orbits with increased muscle volume and those with not-increased muscle volume. Furthermore, the number of cases affected by DON was not significantly higher in the increased group compared with the not-increased group. One might have suspected that there would be a majority of cases with increased muscle volume in a cohort of severe cases requiring surgical treatment, as this seems to be associated with more severe disease [6,7,10,12,26]. However, our results support the hypothesis that the absence of an increased muscle volume does not imply a less severe course or that DON is necessarily associated with an increased muscle volume [12,13]. Nevertheless, muscle diameter and volume at the orbital apex is of particular interest in the context of DON (“apical crowding”) [27,28,29,30]. Several studies have shown a significant difference in apical crowding between orbits with and without DON [5,6,12]. However, an analysis of the functional outcome after surgical therapy depending on these morphological differences is still missing.

In our analysis, the mean preoperative visual acuity was significantly lower in the increased vs. the not-increased group, also in the subgroup of DON-affected orbits. Although the number of orbits affected by DON itself was not significantly different between the increased and the not-increased group, an increased muscle volume nevertheless resulted in significantly worse preoperative visual acuity. Interestingly, however, decompression led to a significant improvement in visual acuity in the increased group, so after surgery there was no longer a significant difference between the mean visual acuity of the increased vs. the not-increased group. Presumably, the lower extent of postoperative improvement of visual acuity in the group with a not-increased orbital muscle volume fraction is related to the fact that preoperative visual acuity was less reduced in this group. In the subgroup of DON-affected orbits, surgical therapy resulted in a significant improvement in visual acuity in both the not-increased and the increased group. Again, the improvement in the increased group was such that the preoperative significant difference in mean visual acuity between the groups was eliminated postoperatively.

Thus, there was a strong association between increased orbital muscle volume and lower visual acuity, and remarkably, there was an excellent response to surgical decompression, even in cases with increased orbital muscle volume. As a predictive factor for DON-affected orbits, increased muscle volume was found to be associated with significantly lower visual acuity, and in view of the good response to surgical therapy, decompression should be considered at an early stage, especially in this group.

Along with varying surgical approaches, several individual factors are discussed as possible reasons for the different outcomes of orbital decompression in GO [31]. Morphological factors seem to be particularly important here, such as the shape and angle of the bony orbit and the anatomy of the venous and lymphatic vessels [31,32,33]. Few studies have investigated factors influencing the visual outcome after orbital decompression in GO, such as duration of the disease, preoperative visual acuity, additional orbital fat reduction, and preoperative degree of exophthalmos [16,34,35,36]. CT-based volumetric analyses showed a positive correlation between exophthalmos reduction and decompression volume [37,38]. A recent study designed a phantom model to measure the pressure relief at the orbital apex depending on the localization and extent of bony decompression comparing different surgical techniques for orbital decompression. This concept seems very interesting and should be further pursued and expanded, as it provides an additional factor that has not yet been considered in the choice of surgical approaches [39].

In our study, the mean preoperative exophthalmos in the increased group (total group as well as subgroup affected by DON) was not significantly higher than in the not-increased group, and decompression resulted in a significant exophthalmos decrease in both groups. This decrease was more pronounced in the not-increased group, which is why the persistent mean postoperative exophthalmos was significantly higher in orbits with increased than with not-increased orbital muscle volume. Hence, no clear association could be established between increased orbital muscle volume and a higher degree of preoperative exophthalmos, but exophthalmos was significantly more reduced after decompression when there was not-increased orbital muscle volume compared with increased orbital muscle volume.

The strengths of our study include the large number of cases, the design having a clear division into two groups based on the determined muscle volume with subsequent standardized surgical treatment, and the objective outcome parameters. Limitations arise in the retrospective design, and the need to exclude cases in the absence of appropriate preoperative imaging or missing outcome data. Furthermore, in a retrospective analysis of over 20 years, surgeon-dependent differences may naturally occur despite the standardized surgical procedure, which could have an influence on the decompression volume. This needs to be further investigated in subsequent studies.

## 5. Conclusions

The development of dysthyroid optic neuropathy in Graves’ orbitopathy is not necessarily related to orbital muscle enlargement. Increased orbital muscle volume is associated with a significant reduction in preoperative visual acuity, but not with increased preoperative exophthalmos. An excellent improvement in visual acuity can be achieved by pterional orbital decompression, even with increased orbital muscle volume. Therefore, this factor should be addressed during treatment decisions, and early surgical therapy should be considered particularly in DON-affected cases with orbital muscle enlargement.

## Figures and Tables

**Table 1:** Classification and demographic data of the “not-increased orbital muscle volume” and the “increased orbital muscle volume” group.

		Not-Increased Muscle Volume	Increased Muscle Volume
number of orbits		*n* = 42	*n* = 41
mean muscle volume (in relation to total orbital volume)		15.60% (SD 2.16, SEM 0.33, CI 14.93–16.28)	29.63% (SD 4.00, SEM 0.62, CI 28.36–30.89)
mean age (years)		55.33 (SD 11.08)	58.90 (SD 10.46)
gender	female	29 (69.05%)	23 (56.10%)
	male	13 (30.95%)	18 (43.90%)
side	left	22 (52.38%)	20 (48.78%)
	right	20 (47.62%)	21 (51.22%)
indication for surgery	DON	30 (71.43%)	33 (80.49%)
	cosmetic/functional	12 (28.57%)	8 (19.51%)
EUGOGO classification	moderate-to-severe	12 (28.57%)	8 (19.51%)
	sight-threatening	30 (71.43%)	33 (80.49%)
CAS	active	35 (83.33%)	38 (92.68%)
	inactive	7 (16.67%)	3 (7.32%)
diplopia	preoperatively	28 (66.67%)	29 (70.73%)
	postoperatively	26 (61.90%) (new after surgery: 2)	19 (46.34%) (new after surgery: 0)

SD, standard deviation; SEM, standard error of mean; CI, confidence interval; DON, dysthyroid optic neuropathy; EUGOGO, European group on Graves’ orbitopathy; CAS, clinical activity score; postoperatively, within the six months after surgery.

**Table 2:** Intra- and inter-group statistical analysis of pre- and postoperative visual acuity (analysis within and between “not-increased” and “increased” orbital muscle volume).

		Not-Increased Muscle Volume	Increased Muscle Volume	Inter-Group Comparison: Change in VA (*p*-Value)
				
**total group** **(DON + cosm/func)**	no. of orbits	*n* = 35	*n* = 40	
	mean VA pre	0.28 logMAR {0.53 decimal} (SD 0.42, CI 0.14–0.43)	0.53 logMAR {0.30 decimal} (SD 0.59, CI 0.34–0.72)	***2.5 lines*** ***(p = 0.0058)***
	mean VA post	0.15 logMAR {0.72 decimal} (SD 0.19, CI 0.08–0.21)	0.36 logMAR {0.44 decimal} (SD 0.57, CI 0.18–0.54)	2.1 lines (*p* = 0.0585)
	change in VA post vs. pre (*p*-value)	1.3 lines (*p* = 0.0761)	***1.7 lines*** ***(p = 0.0138)***	
				
**DON group**	no. of orbits	*n* = 28	*n* = 32	
	mean VA pre	0.34 logMAR {0.46 decimal} (SD 0.45, CI 0.17–0.52)	0.63 logMAR {0.24 decimal} (SD 0.62, CI 0.41–0.85)	***2.9 lines*** ***(p = 0.0035)***
	mean VA post	0.18 logMAR {0.66 decimal} (SD 0.20, CI 0.10–0.25)	0.43 logMAR {0.38 decimal} (SD 0.62, CI 0.21–0.65)	2.5 lines (*p* = 0.0578)
	change in VA post vs. pre (*p*-value)	***1.6 lines*** ***(p = 0.0447)***	***2.0 lines*** ***(p = 0.0142)***	
				
**cosm/func group**	no. of orbits	*n* = 7	*n* = 8	
	mean VA pre	0.04 logMAR {0.92 decimal} (SD 0.10, CI −0.05–0.13)	0.11 logMAR {0.78 decimal} (SD 0.08, CI 0.04–0.18)	0.7 lines (*p* = 0.1834)
	mean VA post	0.04 logMAR {0.92 decimal} (SD 0.08, CI −0.03–0.12)	0.08 logMAR {0.84 decimal} (SD 0.07, CI 0.02–0.13)	0.4 lines (*p* = 0.3930)
	change in VA post vs. pre (*p*-value)	0.0 lines (*p* > 0.9999)	0.3 lines (*p* = 0.5097)	

VA, visual acuity; total group (DON + cosm/func), all cases with available pre- and postoperative assessment of VA in which dysthyroid optic neuropathy (DON) was the indication for surgery or which were operated due to cosmetic/functional reasons; DON group, all cases of “total group” in which DON was the indication for surgery; cosm/func group, all cases of “total group” which were operated due to cosmetic/functional reasons; no., number; vs., versus; change in VA post vs. pre, difference in post- vs. preoperative VA; logMAR, logarithm of the mean angle of resolution; pre, preoperatively; post, postoperatively (within six months after surgery); SD, standard deviation; CI, confidence interval; bold italic font, significant difference.

**Table 3:** Intra- and inter-group statistical analysis of pre- and postoperative exophthalmos (analysis within and between “not-increased” and “increased” orbital muscle volume).

		Not-Increased Muscle Volume	Increased Muscle Volume	Inter-Group Comparison: Change in Exoph (*p*-Value)
				
**total group** **(DON + cosm/func)**	no. of orbits	*n* = 31	*n* = 30	
	mean exoph pre (in mm)	23.16 (SD 1.88, CI 22.47–23.85)	24.40 (SD 4.04, CI 22.89–25.91)	1.24 mm (*p* = 0.1140)
	mean exoph post (in mm)	19.77 (SD 2.46, CI 18.87–20.67)	21.48 (SD 3.59, CI 20.14–22.82)	***1.71 mm (p = 0.0072)***
	change in exoph post vs. pre (*p*-value)	***3.39 mm*** ***(p < 0.0001)***	***2.92 mm*** ***(p = 0.0082)***	
				
**DON group**	no. of orbits	*n* = 24	*n* = 24	
	mean exoph pre (in mm)	23.38 (SD 1.81, CI 22.61–24.14)	24.17 (SD 4.39, CI 22.31–26.02)	0.79 mm (*p* = 0.4564)
	mean exoph post (in mm)	19.83 (SD 2.64, CI 18.72–20.95)	21.04 (SD 3.86, CI 19.41–22.67)	1.21 mm (*p* = 0.1166)
	change in exoph post vs. pre (*p*-value)	***3.55 mm (p < 0.0001)***	***3.13 mm (p = 0.0309)***	
				
**cosm/func group**	no. of orbits	*n* = 7	*n* = 6	
	mean exoph pre (in mm)	22.43 (SD 2.07, CI 20.51–24.34)	25.33 (SD 2.16, CI 23.07–27.60)	***2.90 mm (p = 0.0408)***
	mean exoph post (in mm)	19.57 (SD 1.81, CI 17.90–21.25)	23.25 (SD 1.08, CI 22.11–24.39)	***3.68 mm (p = 0.0058)***
	change in exoph post vs. pre (*p*-value)	***2.86 mm (p = 0.0262)***	2.08 mm (*p* = 0.0909)	

exoph, exophthalmos; total group (DON + cosm/func), all cases with available pre- and postoperative assessment of exophthalmos in which dysthyroid optic neuropathy (DON) was the indication for surgery or which were operated due to cosmetic/functional reasons; DON group, all cases of “total group” in which DON was the indication for surgery; cosm/func group, all cases of “total group” which were operated due to cosmetic/functional reasons; no., number; pre, preoperatively; post, postoperatively (within the six months after surgery); vs., versus; change in exoph post vs. pre, difference in post- vs. preoperative exophthalmos; SD, standard deviation; CI, confidence interval; bold italic font, significant difference.

## Data Availability

The data used to support the findings of this study are available from the corresponding author upon request.
